# From perceptual to lexico‐semantic analysis—cortical plasticity enabling new levels of processing

**DOI:** 10.1002/hbm.22939

**Published:** 2015-08-25

**Authors:** Lara Schlaffke, Naima N. Rüther, Stefanie Heba, Lauren M. Haag, Thomas Schultz, Katharina Rosengarth, Martin Tegenthoff, Christian Bellebaum, Tobias Schmidt‐Wilcke

**Affiliations:** ^1^ Department of Neurology BG‐University Hospital Bergmannsheil, Ruhr‐University Bochum Bochum Germany; ^2^ Department of Neuropsychology Ruhr‐University Bochum Bochum Germany; ^3^ Department of Computer Science University of Bonn Germany; ^4^ Department of Experimental Psychology University of Regensburg Germany; ^5^ Department of Psychology Heinrich‐Heine University Düsseldorf Germany

**Keywords:** levels of processing, fMRI, learning, cortical plasticity, perception

## Abstract

Certain kinds of stimuli can be processed on multiple levels. While the neural correlates of different levels of processing (LOPs) have been investigated to some extent, most of the studies involve skills and/or knowledge already present when performing the task. In this study we specifically sought to identify neural correlates of an evolving skill that allows the transition from perceptual to a lexico‐semantic stimulus analysis. Eighteen participants were trained to decode 12 letters of Morse code that were presented acoustically inside and outside of the scanner environment. Morse code was presented in trains of three letters while brain activity was assessed with fMRI. Participants either attended to the stimulus length (perceptual analysis), or evaluated its meaning distinguishing words from nonwords (lexico‐semantic analysis). Perceptual and lexico‐semantic analyses shared a mutual network comprising the left premotor cortex, the supplementary motor area (SMA) and the inferior parietal lobule (IPL). Perceptual analysis was associated with a strong brain activation in the SMA and the superior temporal gyrus bilaterally (STG), which remained unaltered from pre and post training. In the lexico‐semantic analysis post learning, study participants showed additional activation in the left inferior frontal cortex (IFC) and in the left occipitotemporal cortex (OTC), regions known to be critically involved in lexical processing. Our data provide evidence for cortical plasticity evolving with a learning process enabling the transition from perceptual to lexico‐semantic stimulus analysis. Importantly, the activation pattern remains task‐related LOP and is thus the result of a decision process as to which LOP to engage in. *Hum Brain Mapp 36:4512–4528, 2015*. © **2015 The Authors. Human Brain Mapping Published byWiley Periodicals, Inc**.

**Abbreviations**
BA, Brodmann Area;
FWE: family wise error
IFC: inferior frontal cortex
IPL: inferior parietal lobule
ITC: inferior temporal cortex
LOP: levels of processing
LOSGO: leave one subject per group out
MC: Morse Code
OTC: occipitotemporal cortex
SMA: supplementary motor area
SEM: standard error of means
STG: superior temporal gyrus
STS: superior temporal sulcus;
VWFA: visual word form area

## INTRODUCTION

On a neural level, sensory information undergoes extensive processing and attentional modulation as it becomes incorporated into the texture of perception and cognition [Mesulam, 2008, 1998]. The neural mechanisms underlying both perceptive evaluation and associative elaboration of a given stimulus and the transition from the one to the other still remain to be fully elucidated [Sathian et al., 2013; Trachtenberg et al., 2002; Turennout et al., 2000]. Learning and/or training can either improve an existing skill, enabling a faster and/or more accurate stimulus analysis (e.g., perceptual learning), or it can lead to the acquisition of a new skill accompanied by a shift in the underlying cognitive process, which in turn enables a change in levels of processing (LOPs).

Traditionally, LOPs have been studied in the context of episodic memory research, where the LOP effect describes a very robust and well‐replicated behavioral phenomenon. Specifically, attending to the meaning of a stimulus (semantic; deep processing) is associated with a better retention of the episodes compared to attending to the perceptual aspects of the same stimulus (shallow processing; for a review see Craik, 2002). LOP effects can be very large, and, importantly, they can also occur in the absence of any intention to encode the episode [Craik and Lockhart, 1972; Craik and Tulving, 1975; Kapur et al., 1994]. As such, encoding seems to be a by‐product of processing, but current research is attempting to disentangle neural mechanisms of processing and encoding. Due to the primary interest in episodic memory formation, LOPs have been investigated mainly for skills and/or knowledge already present when performing the task. A typical task would e.g., involve the distinction between living and nonliving objects (word animacy task−deep processing) and judging the number of syllables in a word (shallow processing).

LOPs have been investigated in a number of brain imaging studies, including both activation as well as connectivity analyses, which have begun to shed light on the underlying neural correlates [Rose et al., 2015]. Using PET imaging, Kapur et al. [1994] was the first to demonstrate an increased activation in the left inferior frontal cortex (IFC) during semantic analysis as compared to perceptual analysis [Kapur et al., 1994]. Otten et al. [2001] using fMRI, found greater activation in the medial frontal gyrus, the ventral extent of the left IFC and the left parahipppcampal gyrus in a word animacy task as compared to the corresponding syllable task [Otten and Rugg, 2001]. More recently, connectivity analyses have been performed to investigate the interaction of various brain regions at different LOPs. Schott et al. [2013] could demonstrate differences in LOP‐related hippocampal connectivity, suggesting different roles for the left and right hippocampus for shallow and deep processing.

However, despite the progress that has been made in LOP research, imaging studies in this field are usually cross‐sectional in their design, i.e., are performed at a given time point, and little attention has been paid to the pre‐existing skills and knowledge, in particular their acquisition, that enables a switch in processing. In contrast, numerous studies have been performed that have specifically looked at dynamic changes over time in human brain morphology, function and connectivity associated with different types of learning, e.g. motor learning [Draganski et al., 2004], perceptual learning [Frank et al., 2014; Ilg et al., 2008; Sathian et al., 2013], and semantic learning [Draganski et al., 2006; Schmidt‐Wilcke et al., 2010; Stein et al., 2012]. These studies closely looked at pre‐existing skills and tried to relate learning/practice induced changes in performance to changes in brain morphology or function. Different terms, such as *neuroplasticity, cortical plasticity, or reorganization,* have been used to capture the notion that the brain adapts to new environmental demands on different levels, ranging from the synaptic level to the neural circuitry of whole brain networks, enabling the optimization of behavior and performance. For functional brain imaging, Kelly and Garavan [2005] suggested to classify changes in neural activity associated with learning as either a redistribution or a reorganization (proper). According to this classification, redistribution consists of a combination of increases and decreases in existing regional brain activity, whereas reorganization (proper) comprises a recruitment of novel activation sites and a shift in the cognitive process underlying task performance [Poldrack, 2000]. The latter implies that different tasks are being performed at the beginning and at the end of the learning process [Debaere et al., 2004; Fletcher et al., 1999]. Arguably, a shift in a cognitive process is in some way based on a switch in processing. However, to our knowledge, no imaging studies thus far have investigated both neural correlates of learning that enable a cognitive shift and neural correlates of different LOPs once the learning process has been accomplished.

Using Morse code (MC), a method of transmitting text information as a series of on‐off tones, clicks, or lights with different durations (short and long), we sought to investigate both cognitive shifts as well as switches in LOPs. MC can be presented in the context of a perceptual task, e.g., aiming at the distinction between short and long signals, or in the framework of a lexical task, e.g., aiming at the assignment of a specific letter to a certain sound pattern (pattern‐to‐letter/phoneme conversion). Within the learning process a link is establish between an acoustic pattern and an already existing letter representation system, which encompasses both visual as well as phonological letter representations. In this regard learning MC is comparable to learning how to read (a modern Western language) where children learn to decode text based on (a) the understanding that spoken words are made up of phonemes (phoneme awareness), (b) the familiarity with letters (letter knowledge) and (c) the knowledge that letters in written words represent phonemes (alphabetic principle). Decoding text is also assisted by the ability to sound out regular words (cipher knowledge) or the ability to correctly pronounce irregular words (lexical knowledge) [Hoover and Gough, 1990]. While the skilled reader decodes written language rapidly by transforming a set of letters into a visual word form, children at an early stage often apply a letter‐by‐letter reading technique converting consecutive single letters into phonemes [Perfetti and Bolger, 2004]. In this regard, learning MC resembles early reading, as words are processed letter‐by‐letter. In the following, we will refer to this pattern‐to‐letter conversion as lexical analysis and to the corresponding learning process as lexical learning. Since the decision of whether a train of letters makes up a word or not requires additional semantic processing, we will correspondingly refer to this as semantic analysis. Of note in linguistics lexical knowledge refers to the ability to sound out words, even if they do not follow the conventional letter ‐ phoneme relationship [Hoover and Gough, 1990], while lexical identification often refers to the assignment of a word to the sensory input (chosen from a vocabulary of tens of thousands of words); both terms should not be confused with the term lexical analysis as used in this study, which operates on the level of letters as opposed to words or morphemes.

Specifically we sought to address two questions. On the one hand, it is unclear whether different LOPs rely on different patterns of brain activation, or whether a higher LOP just recruits additional brain regions as an add‐on to the brain regions already engaged in the lower LOP. This might be addressed more accurately when looking at dynamic changes associated with the emergence of a new LOP. On the other hand, little is known about the brain activations associated with a lower LOP, once a higher LOP and its new activation pattern have been established. As such, it is conceivable that all LOPs elicit the new activation pattern, or alternatively, that the activation remains very much dependent on the LOP applied. Using MC as a model for learning and processing this study tried to investigate both dynamic changes in brain activation over time and LOP‐related adaption in brain activity. We hypothesized that the analysis of MC depends on a core network and that depending on the LOP different brain regions are additionally recruited to serve specific aspects of the task, i.e. perceptual or lexico‐semantic analysis. Specifically for the lexico‐semantic task we hypothesized that within the learning process a new network would evolve, including regions known to be critically involved in lexical processing, such as the left occipitotemporal (OTC) and inferior temporal cortex (ITC), that would not be recruited within the perceptual task [Nakamura et al., 2004; Poeppel et al., 2012].

## METHODS

### Subjects

Data were collected from 18 healthy, young, right‐handed study participants (10 w, mean age 23 ± 2 years) with inconspicuous T1‐weighted MR images. All subjects were naïve to MC prior to the study. All participants gave written informed consent prior to study enrolment. The study was conducted in accordance with the Declaration of Helsinki and approved by the Ethics Committee of the Faculty of Psychology of Ruhr‐University Bochum, Germany.

### Task and Training

Morse code is a method of transmitting text information as a series of on‐off tones, clicks, or lights. The International Morse Code encodes the ISO basic Latin alphabet, some extra Latin letters, the Arabic numerals and a small set of punctuation and procedural signals as standardized sequences of short and long signals pictured as “dots” (•) and “dashes” (**—)** (the duration of a dash is three times the duration of a dot). Using a subset of 12 letters (A, D, E, G, I, M, N, O, R, S, T, U) at a standardized presentation speed, a highly controlled learning environment was set up using an audio book. As indicated by pilot testing, this procedure enabled a high percentage of study participants to succeed in learning, yet with different performance levels introducing sufficient variability to allow for correlation analyses between performance and brain activation.

**Figure 1:**
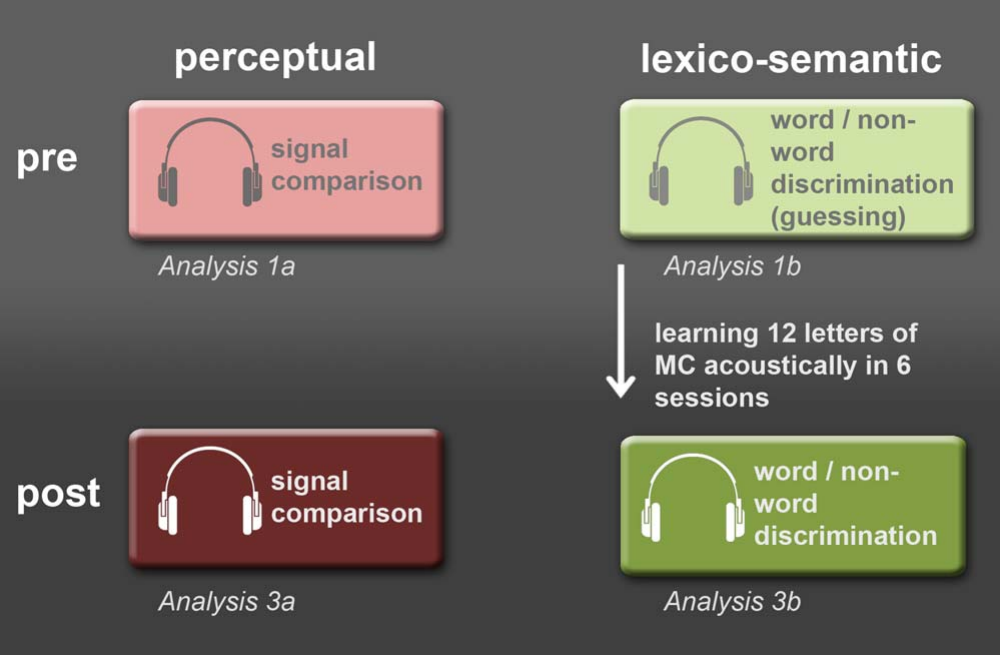
Graphical scheme of the study set up. Decoding sessions were done in the Scanner. Learning period was portioned in six sessions. Each session included repetition of previously learned letters, learning new letters, and practicing 3‐letter decoding. [Color figure can be viewed in the online issue, which is available at http://wileyonlinelibrary.com.]

Study participants learned 12 letters within six supervised learning sessions within 10 days, with an adjournment of one weekend. Within the first training session, four letters (E: •, N: **—** •, O: **— — —**, S • • •) were presented to the participants. Auditory stimuli were presented with varying presentation speeds (between 30 and 60 letters per minute) while participants were asked to decipher the presented Morse code signals and to write down the corresponding letters in a training protocol folder. On the next day, the subsequent training started with a repetition trial of the training session of the preceding day. After the repetition phase, a new training session was started which included two new letters on each subsequent training day (training day 2: R • **—** •, T **—**; training Day 3: D **—** • •, U • • **—**; training Day 4: A • **—**, I • •; training Day 5: G • **— —**, M **— —**). In the last training session (training Day 6), no novel letters were introduced and the audio training only included a repetition of the letters that were previously learned by the participants. At the end of each session, participants performed a word—nonword discrimination task in which they had to indicate in written form, if a presented three‐letter‐train made up a word or a nonword. Only letters already learned were used for this task. All participants underwent two MRI sessions: one before the first training session and one after the last training session (see Fig. 1).

In the scanner, Morse code was presented acoustically in trains of three letters with a total duration of each train between 1.1 and 2.6 s, depending on the respective signals. The three‐letter‐trains made up a word, a nonword or the well‐known SOS signal. Furthermore, a sine wave tone (“Beep”: 786 Hz, 2 s) was presented interleaved with the other stimuli. Participants were taught to decode the SOS signal prior to the first scanning session; both SOS and the Beep sound served as a within session control stimuli (one with and one without a Morse code sound pattern), the processing of which was not expected to depend on a learning process between the two MRI sessions. The three‐letter‐trains of the different conditions were presented in an event‐related design, with a 9 s block and jittered within this block for 0.5, 1, 1.5, 2, or 2.5 s to ensure proper sampling of BOLD‐response. All stimuli were randomized for each session and participant.

**Figure 2:**
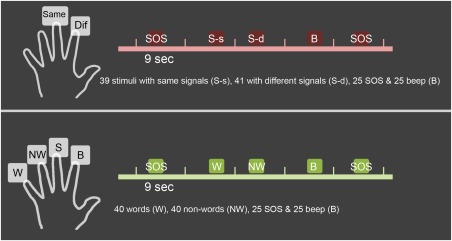
Graphical scheme of the task performed in the scanner. Same stimuli were used for both tasks. All stimuli were presented acoustically and jittered within a 9 s block. B = Beep control sound; nw = nonword (e.g., “dsa” **‐** • • | • • • | • **‐**), S = SOS, w = word (e.g., “gag” **‐ ‐** • | • **‐** | **‐ ‐** •). An example for trains with same signals (long) at the beginning and at the end (S‐s) would be “gut” (“good”) which is encoded as **‐ ‐** • | • • **‐** | **‐** [Color figure can be viewed in the online issue, which is available at http://wileyonlinelibrary.com.]

Each subject underwent the same types of tasks twice in the scanner (two runs pre and two runs post training), using exactly the same set of stimuli. Participants were asked to perform two different tasks, analyzing the tone length of single stimuli (run 1 – perceptual analysis) or meanings (run 2 – lexical analysis). For the perceptual task participants were instructed to analyze the three‐letter‐train and to decide whether the first and last Morse code signal (dot or dash) was the same or not, yielding two different response options (left index and left middle finger, respectively). When the Beep stimulus was presented, participants were instructed to give the answer “same signal” (left index finger), likewise for the SOS stimulus; SOS starts and ends with a short signal: • • • | **— — —** | • • • . In the second run, study participants were then asked to translate the whole train of stimuli and decide whether the letters made up a word (button 1) or a nonword (button 2), or whether it was a SOS signal (button 3) or the control tone (button 4), adding up to four different response options. In the first session before training, participants were not able to translate the letters and were therefore asked to answer intuitively (i.e., to guess) for the words and non‐words. For the SOS and the Beep stimulus participants were instructed to give responses by button press (buttons 3 and 4). They were specifically asked, not to press the buttons 1 and 2 alternately, when words and nonwords were presented. Both runs lasted for 20 min and were paused after 10 min each to give the participant a short rest in the scanner. See Figure 2 for a graphical scheme of the presented tasks.

Responses were recorded during the fMRI sessions and changes in performance both for the perceptual and the lexico‐semantic analysis were assessed using a two‐way ANOVA (factor 1: stimulus anlaysis (perceptual and lexico‐semantic); factor 2: time point). Specifically, we calculated the main effect of time (pre vs. post in both tasks) as well as the interaction (stimulus analysis × time points) to assess differences in task‐related changes. To ensure that participants knew the words that were presented in MC, they were required to read all words and non‐words that were included in the paradigm and indicate whether they were familiar with these words. This was done after the last scanning session.

### fMRI—Sequences

Magnetic resonance imaging (MRI) was performed on a 3.0 Tesla scanner (Philips Achieva 3.2, Best, Netherlands) using a 32‐channel head coil. High‐resolution T1‐weighted data sets (TR 8.3 ms, TE 3.8 ms, FOV 256 × 256, yielding 220 transversal slices with a voxel size of 1.00 × 1.00 × 1.00 mm³ and reconstructed to 0.94 × 0.94 × 1.00 mm³) were acquired first from all subjects. During the actual task, T2*‐weighted echo planar images (single shot EPI with 90° flip angle, TR 2400 ms, TE 35 ms, FOV 224 × 224 mm^2^ with a voxel size of 2 × 2 × 3 mm³ yielding 36 slices in an ascending scan order without gaps) yielding 500 dynamic scans for each task were acquired.

### fMRI—Preprocessing and Statistical Analysis

Functional images for both tasks were transferred from the scanner to a windows work station (Win7, Intel Core i5, 3.2 GHz) and converted from dicom to nifti (hdr‐img pairs) format using MRIconvert 2.0 (Lewis Center for Neuroimaging, University of Oregon). Preprocessing of functional images was performed using SPM8 (Welcome Department of Cognitive Neurology, University College, London, UK) running under Matlab R2008a, (The Mathworks, USA).

The preprocessing steps included slice time correction, unwarping, realignment for movement correction, spatial normalization to the same stereotactic space (using SPM EPI‐template) and spatial smoothing (FWHM: 6 mm).

For both tasks and both scanning sessions statistical analysis of the event related experiment was performed using SPM8. The duration of each stimulus was convolved with the hemodynamic response function (HRF) (Friston et al., 1995). One regressor for each of the four conditions (words, nonwords, SOS and Beep) was modeled for both tasks in the pre and post learning scan sessions, irrespective of response accuracy, yielding 16 contrast‐Images (4 conditions × 2 tasks × 2 time points), which were used for the second level analysis. In this analysis we were not interested in the neural correlates of correct and incorrect answers, i.e., no additional regressors differentiating between right and wrong answers were created. This will be done in a subsequent analysis. As in this study we were also not interested in differences in brain activation elicited by words or nonwords, these two categories were merged. Furthermore six motion parameters (three for rotation and three for translation) were added as nuisance variables to the first level analyses.

In a second level analysis (random effects analysis) contrast images of each condition were analyzed using a flexible factorial design to examine differences between the conditions; a repeated measures ANOVA was performed to compare the different conditions and time points and to create statistical maps. For correction for multiple comparisons see below. For both tasks all presented words and nonwords were contrasted against the Beep serving as a within session control stimulus. The mean reaction time for each condition was added as a nuisance variable. The following analyses (1‐7) were performed:

#### Analysis 1

To investigate baseline brain activation for stimulus presentations before learning, words and nonwords were contrasted with the Beep control stimulus for both tasks (perceptual and lexico‐semantic). In Analysis 1a) we investigated brain activation associated with perceptual analysis pre Morse code learning (pre_perceptual_). In Analysis 1b) we investigated brain activation associated with semantic analysis pre Morse code learning (pre_lexico‐semantic_). Of note, prior to learning study participants were not able to decode any letters of MC; in this sense no lexical and/or semantic analysis could be performed. As such the activation elicited in this task reflects no active work up of the stimuli; this condition depicts a very basic activation pattern serving as a baseline to describe differences pre and post learning.

#### Analysis 2

To cross‐sectionally compare the two different tasks prior to learning, the differences between words/nonwords and Beep were then contrasted between the tasks, yielding the contrasts pre_lexico‐semantic_ > pre_perceptual_ (Analysis 2a) and pre_perceptual_ > pre_lexico‐semantic_ (Analysis 2b).

#### Analysis 3

In analogy to Analysis 1 the baseline brain activations were contrasted post learning to investigate brain activation patterns associated with perceptual analysis after MC learning (Analysis 3a: post_perceptual_) and with semantic analysis post Morse code learning (Analysis 3b post_lexico‐semantic_), each contrasted by brain activation elicited by the Beep.

#### Analysis 4

Cross‐sectional differences between the tasks for time point 2 were calculated in Analysis 4. Brain activation associated more with the semantic task as compared to the perceptual task were contrasted as post_lexico‐semantic_ > post_perceptual_ (Analysis 4a), whereas brain activations associated more with the perceptual task as compared to the semantic task were contrasted as post_perceptual_ > post_lexico‐semantic_ (Analysis 4b).

#### Analysis 5

Learning related changes in brain activation were investigated by longitudinally comparing the differences between words/nonwords and the Beep for both tasks separately. This led to the contrasts post_perceptual_ > pre_perceptual_ (Analysis 5a) and post_lexico‐semantic_ > pre_lexico‐semantic_ (Analysis 5b).

#### Analysis 6

To demonstrate where the changes in brain activation elicited by MC stimuli (as compared to the Beep) increases over time more in the lexico‐semantic task than in the perceptual task, the following contrast was calculated: (post_lexico‐semantic_ > pre_lexico‐semantic_) > (post_perceptual_ > pre_perceptual_). This interaction analysis was performed in order to investigate whether learning as a dynamic process in the lexico‐semantic task would be associated with new activations not seen in the perceptual task.

#### Analysis 7

Finally, for the comparison of perceptual and lexico‐semantic analysis (after learning) and for the comparison of the lexico‐semantic analysis (pre and post learning) we performed multivariate analyses. Specifically, we were interested whether we could train a linear support vector machine (SVM), to differentiate between the two analysis conditions, and identify patterns of brain activation specific to a certain condition. Successful classification provides an additional confirmation of the fact that significant differences exist between the two conditions. Multivariate analyses were performed using the Pattern Recognition for Neuroimaging Toolbox (PRoNTo, Schrouff et al., 2013) and a gray matter mask, yielding a feature vector consisting of 113,480 voxels. Beta images, derived from the perceptual and lexico‐semantic analysis, respectively (words and nonwords) at time point 2, were entered into the analysis (Analysis 7a). In brief, the objective is to derive a data‐based function from the data that can accurately predict the labels, i.e. perceptual or lexico‐semantic analysis. Classification was performed by training a linear SVM (*f*(*x*) = *w*_0_+*w* ^T^x, where the weights *w* are the model parameters learned in the training phase and represent the relative contribution of each feature to the predictive task [Schrouff et al., 2013]. A default regularization parameter *C* = 1 was applied. Classification accuracy was estimated in a leave‐one‐subject‐per‐group‐out manner (LOSGO). In a first step, we tested whether the classifier performed significantly better than chance level (50%), and in a second step we projected the average weight maps back on a brain template to visualize brain regions that contributed most to the classification as “perceptual” or “semantic'”(Figure 6). We applied the same multivariate analysis strategy (LOSGO) to classify beta images, derived from the lexico‐semantic task (words and nonwords) pre and post learning (Analysis 7b).

For the univariate analyses all reported contrasts are thresholded at *P* < 0.05 (whole brain FWE corrected, voxel level, with an extent threshold of *k* = 50 voxel, except Analysis 6 where we allowed a less stringent extent threshold of *k* = 10 voxels (due to the confirmatory nature of this test). Reported coordinates are given in MNI‐coordinates. The analysis of SOS‐related brain activation will be part of future analysis and will not be reported here. Of note, taking SOS as a control stimulus, instead of the Beep, yielded the same results, and will therefore not be reported separately. Anatomical labeling of brain regions showing significant differences between tasks was performed using the SPM8 extension XjView (http://www.alivelearn.net/xjview8/). Visualization of results was performed using MRIcron (http://www.cabiatl.com/mricro/mricron/index.html).

## RESULTS

### Performance

**Figure 3:**
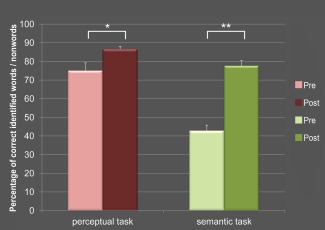
Relative scores and standard error of means (SEM) of task performances; pink: perceptual performance at time point 1, maroon: perceptual performance at time point 2, light green: lexico‐semantic performance at time point 1, green: lexico‐ semantic performance at time point 2. There was a significant increase in performance in both tasks (* = *p* < 0.05; ** = *p* < 0.001). [Color figure can be viewed in the online issue, which is available at http://wileyonlinelibrary.com.]

All participants completed the learning phase and the scanning sessions (no dropouts). There was a significant effect of time (*F*_2,17_ = 86,74; *P* < 0.001). Post hoc *t*‐tests revealed that study participants performed significantly better in both, the perceptual and lexico‐semantic tasks in the second run as compared to the first, showing a gain from 74.6% (SEM ± 4.25) to 86% (SEM ± 3.04, *P* < 0.05) in the perceptual task and a gain of 42.6% (SEM ± 1.45) to 76.9% (SEM ± 2.80, *P* < 0.001) in the lexico‐semantic task. The interaction analysis revealed a significant interaction between time and task (*F*_2,17_ = 19.38, *P* < 0.001), i.e. study participants improved significantly more in the lexico‐semantic task as compared to the perceptual task. The given values are relative scores of correct answers, i.e., same/different for the perceptual task, and words/nonwords for the semantic task (sum of correctly identified words and nonwords divided by all words and nonwords, see also Figure 3).

### Brain Imaging

#### Analysis 1a (A‐1a)

**Figure 4:**
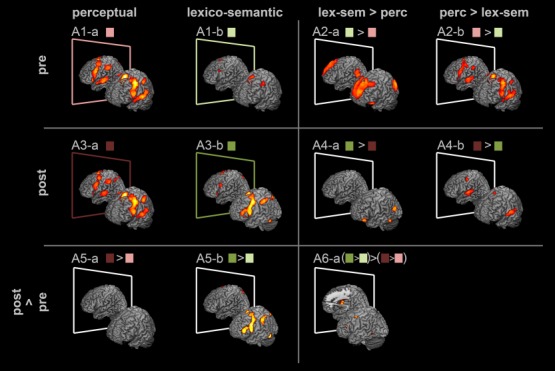
Cortical brain activation of the tasks (words and nonwords > beep) overlaid on an MNI‐template. Left hemisphere is shown, right hemispheric activations are displayed in mirrors. Of note, for display purposes brains are presented from slightly different viewing angles. [Color figure can be viewed in the online issue, which is available at http://wileyonlinelibrary.com.]

Comparing all words and nonwords in the perceptual task (stimuli with same or different signals at the beginning and the end) to the Beep control stimulus, we found activations in the supplementary motor area (SMA), the insula, the primary auditory cortex, the inferior frontal gyrus and the inferior parietal lobule (IPL, BA 40) and one cluster in the left superior parietal lobule (BA 7/precuneus). For details see Figure 4(A‐1a) and Table 1.

**Table 1:** Analysis 1 and 2a

Pre learning																					
			Allwords > Beep Perceptual Task						Allwords > Beep lexico‐semantic Task						lexico‐semantic Task > Perceptual Task						
			Refers to analysis 1‐a						Refers to analysis 1‐b						Refers to analysis 2‐a						
			*x*	*Y*	*z*	*t*‐value	Voxel #	BA	*x*	*Y*	*z*	*t*‐value	Voxel #	BA	*X*	*y*	*z*	*t*‐value	Voxel #		BA
Frontal lobe	Superior Frontal Gyrus	L	0	12	52	14.55	1197	6/SMA	2	22	43	9.02	544	6/SMA	−12	60	22	9.61	3800		10
		L													−10	48	46	9.46	in 3800		8
Middle Frontal Gyrus		L							−50	0	52	6.81	82	6/premotor cortex							
Inferior Frontal Gyrus		R	34	24	−2	9.00	213	Insula	50	12	37	5.61	55	9							
		L	−30	24	−2	8.60	219	Insula							−44	30	−17	8.35	165		47
		L	−56	8	19	9.69	in1710	44													
		R	46	8	31	10.42	1271	6													
		L	−54	6	40	11.07	in1710	9													
Precentral Gyrus		L	−48	−2	49	12.98	1710	6													
Middle Temporal Gyrus		L													−46	−70	34	9.76	1074		39
		R													54	−70	22	8.40	635		39
Superior Temporal gyrus		L	−64	−20	4	11.09	879	22/prim. auditory													
		R	62	−26	1	9.38	858	22/prim. auditory													
Parietal Lobe	Inferior Parietal Lobule	R	44	−40	46	8.57	483	40													
		L	−44	−40	46	9.59	493	40							−54	−62	37	8.24	in 1074		40/TPJ
Precuneus		L	−16	−70	49	7.01	82	7							−4	−36	37	8.60	1444		31
Limbic Lobe	Cingulum														0	−14	40	7.18	165		0
Parahippocampal Gyrus		L														−30	−30	−17	6.80	61	0

MNI‐coordinates (*x y z*) of peak voxel and cluster sizes of prelearning activations. For clusters with more than one peak, cluster sizes are reported as “in”. BA, Brodmann‐area.

#### Analysis 1b (A‐1b)

Comparing the same stimuli, words and nonwords to the Beep in the lexico‐semantic task we found a cluster in the SMA and additional clusters in the left precentral gyrus (BA 6) and the right inferior frontal gyrus (BA9). See Figure 4 (A‐1b).

#### Analysis 2a (A‐2a)

When comparing pre_lexico‐semantic_ > pre_perceptual_ we found activations in the precuneus, the medial frontal gyrus region and the temporo‐parietal junction bilaterally. See Figure 4(A‐2a) and Table 1. Since these regions are major components of the default mode network, which is typically deactivated when a task is being performed, we then extracted the signal intensities of the clusters. Relative signal increases during the lexico‐semantic task as compared to the perceptual task are based on less deactivation in the lexico‐semantic task.

#### Analysis 2b (A‐2b)

However, in the opposite contrast, when comparing pre_perceptual_ > pre_lexico‐semantic_ we found a similar network as for the perceptual task itself including activation in the supplementary motor area (SMA), the insula, the primary auditory cortex, the middle frontal gyrus and the inferior parietal lobule (BA 40). For details of Analyses A‐1 and A‐2 (peak coordinates and cluster extents) see Table 1).

#### Analysis 3a (A‐3a)

After completing the learning phase the perceptual task (post_perceptual_) yielded the same activation pattern as the pre learning condition (pre_perceptual_). The SMA was activated as well as the STG, the insula, the middle frontal gyrus and the inferior parietal lobule (BA 40) bilaterally. See Figure 4(A‐3a) and Table 2.

**Table 2:** Analysis 3 and 4a

Post learning																				
			Allwords > Beep Perceptual Task						Allwords > Beep lexico‐semantic Task						lexico‐semantic Task > Perceptual Task					
			refers to analysis 3‐a						refers to analysis 3‐b						refers to analysis 4‐a					
			*x*	*y*	*z*	*t*‐value	Voxel #	BA	*x*	*y*	*z*	*t*‐value	Voxel #	BA	*x*	*y*	*z*	*t*‐value	Voxel #	BA
Frontal Lobe	Superior Frontal Gyrus	L	0	12	52	15.31	1169	6/SMA	−2	10	58	11.47	783	6/SMA						
	Middle Frontal Gyrus	R	30	2	55	9.38	372	6/premotor cortex												
		L	−46	0	52	13.16	1802	6/premotor cortex	−48	0	52	10.76	1880	6/premotor cortex						
	Inferior Frontal Gyrus	R	44	6	31	9.12	548	6/premotor cortex	46	8	31	7.34	105	6						
		R	34	24	−2	8.18	108	Insula	34	24	−5	7.45	98	Insula						
		L	−32	22	1	8.40	137	Insula	−30	22	2	7.91	164	Insula						
		L							−52	8	37	9.79	in 1880	9	−32	34	−14	6.55	125	47
Temporal Lobe	Inferior Temporal Gyrus	L							−54	−58	−17	7.32	71	20/FF	−48	−50	−20	6.40	107	37/FF/OTC
	Middle Temporal Gyrus	L													−44	−74	25	6.61	119	39
	Superior Temporal gyrus	L	−62	−20	4	9.56	700	22/prim. auditory												
		R	66	−22	7	8.04	524	22/prim. auditory												
Parietal Lobe	Inferior Parietal Lobule	R	42	−40	43	8.86	584	40												
		L	−46	−38	49	10.29	964	40	−46	−38	49	8.13	812	40						

MNI‐coordinates (*x y z*) of peak voxel and cluster sizes of post‐learning activations. For clusters with more than one peak, cluster sizes are reported as **“**in**”**. BA, Brodmann‐area.

#### Analysis 3b (A‐3b)

The lexico‐semantic task (post_lexico‐semantic_) yielded a new activation pattern after learning. In addition to the SMA, the right inferior frontal gyrus and the left precentral gyrus and the insula were activated bilaterally as well as the left inferior parietal gyrus (BA40), the superior parietal gyrus (BA7), a large cluster in the left inferior frontal cortex (including BA 44/45) and the inferior temporal gyrus (BA20/fusiform gyrus). For details see Table 2 and Figure 4(A‐3b).

#### Analysis 4a (A‐4a)

**Figure 5:**
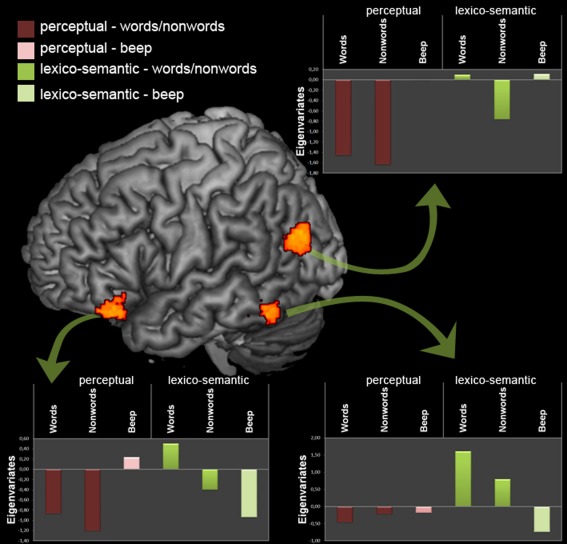
Brain activations of post **lexico‐**semantic_(words/nonwords > beep)_ > perceptual_(words/nonwords > beep)_ (Analysis 4a) overlaid on the MNI‐ template. Bar graphs show the eigenvariates of the respective clusters separated in conditions. The same color code as in Figure 3 was used. [Color figure can be viewed in the online issue, which is available at http://wileyonlinelibrary.com.]

The between task comparison post learning for the analysis post_lexico‐semantic_ > post_perceptual_ revealed clusters in the left occipitotemporal cortex (OTC) (including both the fusiform gyrus and left inferior temporal gyrus) the left IFC, as well as the left middle temporal gyrus. To demonstrate the contribution of the conditions (word/nonwords and Beep, during the perceptual and semantic analysis, pre‐ and post learning), we extracted the eigenvariates from the clusters and plotted the corresponding bar graphs (see Figure 5). For the cluster in the left OTC both the activation elicited by words and nonwords (as compared to the Beep) at time point 2 contributed to the contrast, while for the left IFC it was mainly the activation elicited by words.

#### Analysis 4b (A‐4b)

Contrasting post_perceptual_ > post_lexico‐semantic_ yielded activation clusters in the SMA and the STG (including the primary auditory cortices) bilaterally (see Figure 4 and Table 2).

#### Analysis 5a (A‐5a)

Longitudinal analysis revealing learning related changes in brain activation patterns over time did not show any significant results in the perceptual task when comparing the differences between words/nonwords and Beep post_perceptual_ > pre_perceptual_ (see Figure 4).

#### Analysis 5b (A‐5b)

When analyzing learning related changes in the lexico‐semantic task by contrasting post**_lexico‐_**_semantic_ > pre_lexico‐semantic_, we found almost the same activation pattern as for the basic contrast post_lexico‐lemantic_ (see analysis 3b) except from the cluster in right BA9) but with additional clusters in the caudate head bilaterally See Figure 4(A‐5b).

#### Analysis 6 (A‐6a)

The contrast (post_semantic_ > pre_lexico‐semantic_) > (post_perceptual_ > pre_perceptual_) revealed significant activation clusters in the caudate nuclei bilaterally (peak coordinates left x = −10, y = 8, z = 28, k = 310 voxels; right x = 18, y = 12, z = −5, k = 425 voxels), the left middle/inferior temporal gyrus (peak coordinates x = −44, y = −56, z = −2, k = 11 voxels), the left inferior frontal gyrus (peak coordinates x = −40, y = 26, z = 16, 35 voxels) the left precentral gyrus (BA 6, peak coordinates x = −50, y = −6, z = 52, 20 voxels) and in the supplementary motor area (SMA peak coordinates x = −6, y = 0, z = 61, 45 voxels).

#### Analysis 7a (A‐7a)

**Figure 6:**
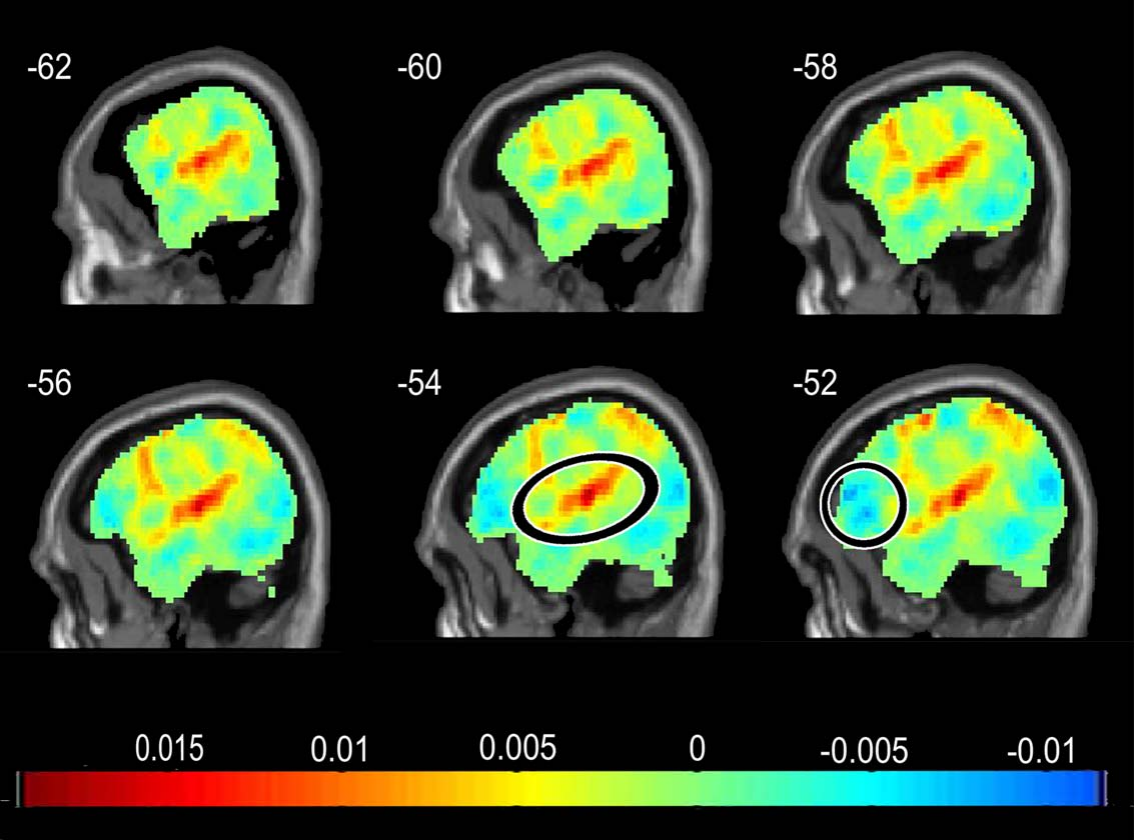
Coefficients of the weight vector of the SVM analysis. Activations in regions with a strong negative weight (shown in blue) led to classification as “lexico‐semantic analysis”, in regions with a positive weight (shown in red), they led to classification as “perceptual analysis”. [Color figure can be viewed in the online issue, which is available at http://wileyonlinelibrary.com.]

In the multivariate analysis of the post learning conditions (post_perceptual_ and post_lexico‐semantic_), 32 out of 36 images were classified correctly as being perceptual or semantic (accuracy: 89%, chance level at 50%). A permutation test rejected the null hypothesis that this result was obtained by chance (p = 0.002). Visual inspection of the weight vector *w*, used by the support vector machine to distinguish between the conditions, indicates that multivariate analysis relied on regions similar to the ones found by univariate testing. In particular, the signs of the weight vectors were such that activation in the STG bilaterally and the SMA contributed to the classification as perceptual analysis, while activations in the left IFG and the left ITC, but also in the caudate nuclei, the temporal pole and the precuneus contributed to classification as semantic analysis (Figure 6).

#### Analysis 7b (A‐7b)

In the multivariate analysis of the semantic conditions (pre_lexico‐sematic_ and post_lexico‐semantic_), 28 out of 36 images were classified correctly as being pre or post learning (accuracy: 78%, chance level at 50%). A binomial test rejected the null hypothesis that this result was obtained by chance (p = 0.006). In particular, the signs of the weight vectors were such that activation in the right premotor/motor cortex contributed to the classification as pre_lexico‐semantic_ analysis, while activations in the left IFG and the left ITC, but also bilaterally in the hippocampus contributed to classification as post_lexico‐semantic_ analysis.

## DISCUSSION

In this study we sought to investigate the transition from perceptual to lexico‐semantic stimulus analysis associated with a newly acquired skill, i.e. the ability to decode MC. Given that the primary auditory cortex serves as the sensory input channel to the cortex in both tasks, but that different tasks require the engagement of different networks, we were interested in determining how the network involved in the lexico‐semantic task would evolve within the learning process and, moreover, to what degree the networks recruited by the perceptual and lexico‐semantic tasks would differ. More specifically, we were interested in differences in activation related to the LOP (A4‐a and A4‐b) and changes over time related to learning and cortical plasticity (A5‐b).

As a main result, we report that both tasks engaged a core network consisting of the left premotor cortex, the SMA, and the left inferior parietal lobule (IPL) and that depending on the task either the superior temporal gyrus (STG) bilaterally for the perceptual task or the left OTC, as well as the left IFG for the lexico‐semantic task showed task specific activations. The learning process on the other hand, comparing the lexico‐semantic task pre and post learning, was associated with the activation of the core network, the left OTC and the left IFG. While the activation of the core network is not specific to the lexico‐semantic task and can also be observed in the perceptual task at baseline, we propose that the new activation seen in the left OTC and the left IFG are closely related to the cognitive shift underlying the new ability to decode MC and in this regard reflects cortical reorganization and plasticity. Of note, also increases in activations in the caudate nuclei were observed in the lexico‐semantic task. While we appreciate that this is an interesting finding, possibly related to procedural memory, we were more interested in cortical networks in this study and will discuss the caudate activations elsewhere. From here we will refer to the different cortical networks as the “core”, the “perception” and the “lexico‐semantic” network, respectively.

## CORE NETWORK

Our results provide strong evidence for a mutual core network, involved in both tasks, consisting of the left premotor cortex, the SMA, and the left IPL. This core network was activated by the perceptual task (pre and post learning) and by the lexico‐semantic task (post learning). In the lexico‐semantic task pre learning only small activation clusters in the SMA and the right inferior frontal gyrus were observed. Importantly, for the lexico‐semantic task at baseline (pre learning) no comparison between any signals/letters had to be performed and as such no memory load was created. Against this background, we hypothesize that the core network is involved in working memory. Specifically for the premotor cortex, it is conceivable that its strong activation might also be related to rhythm perception [Grahn et al., 2011], prediction of sequential perception [Schubotz et al., 2000; Schubotz and von Cramon, 2002] or internal sound production [Price et al., 2011]. For example, short and long signals in the perceptual task are assigned “dit” and “dah” sounds, respectively. After the learning process, the compilation of internally‐created sounds (letters) may then also help to perform the lexico‐semantic task. However, in the case of internal sound production, bilateral activation would be expected, which was only observed in the perceptual task. This needs to be further investigated in future studies.

## LEVELS OF PROCESSING

Depending on the LOP, when comparing the perceptual analysis and the lexico‐semantic analysis (after learning) either primary sensory cortices or higher association cortices were engaged to handle specific task requirements. The perceptual task was associated with a strong activation in the SMA as well as in the STG bilaterally, encompassing the primary auditory cortex and the posterior part of the temporal sulcus (pSTS). The activation in the bilateral STG was significantly stronger in the perceptual task as compared to the lexico‐semantic task and did not change significantly over time (of note, during lexical learning no specific perceptual training was performed). The lexico‐semantic task on the other hand was associated with additional activations in the left OTC including the fusiform gyrus, and in the left ITC including Broca's area, after learning. Significantly less activation was seen in the STG during the lexico‐semantic as compared to the perceptual analysis.

The STG, comprising the primary auditory cortex and the STS, plays a major role in spectrotemporal analysis in early sound processing [Hickok and Poeppel, 2007], but is also known to serve as a unimodal (auditory) association area [Goldberg et al., 2006]. Still at the prelexical stage the auditory input is then processed along an antero‐lateral pathway (ventral stream). The anterior part of the STG plays a role in basic object recognition, e.g. the distinction between speech and environmental sounds [Rauschecker and Scott, 2009], whereas the posterior part of the STG seems to be more involved in processing spatial information (dorsal stream) [Kreher et al., 2008]. Interestingly, strong activation in the STG was seen during the perceptual task only, indicating an important role of this region in the processing of basic sound features and related decision making.

The activation cluster in the left OTC seen during the semantic analysis only, was mainly located in the left inferior temporal gyrus and extended to the left fusiform gyrus as well as to the left lateral occipital gyrus. This region is of specific relevance to this study for several reasons. First, in the context of (aural) speech processing, it has been suggested that the middle and inferior portions of the temporal lobe, as part of the ventral processing stream, serve as a lexical interface that link phonological to semantic information [Hickok and Poeppel, 2007]. Second, the activation cluster also extended into the visual word form area (VWFA). Although it is still a matter of debate whether such a highly specialized region for visual word identification actually exists [Kronbichler et al., 2004; Price and Devlin, 2003], this region has repeatedly been shown to be critically involved in the identification of written words. Some authors have even suggested that a subpart of the left fusiform gyrus in BA 37 might be specific to letter processing [Flowers et al., 2004; Pernet et al., 2005; Polk and Farah, 1998], suggesting that the fusiform gyrus sustains access to letter representation in memory. Nonetheless, as pointed out, the fusiform gyrus and the VWFA have been viewed as the part of the visual association cortex primarily involved in decoding written language, particularly involving early alphabetic processing within the visual ventral stream. Our data provide evidence that specific sound patterns can elicit brain activation in this region and we argue that this activation is involved in sound pattern to letter conversion. We hypothesize that during the learning process the STG functionally connects to the left OTC, a process that may well be supported by the practicing strategy where subjects were required to write down letters after decoding them, providing a visual control. This would be an example of a cross‐modal analysis strategy, where a unimodal stimulus gains lexical access to a kind of knowledge (letter recognition) that in the personal history of the study subject has predominantly been acquired through visuo‐motor training. Finally in a previous study we found an increase in gray matter density in participants learning Morse code (the whole alphabet and numbers) [Schmidt‐Wilcke et al., 2010]. However, it remains unclear what exactly the neural underpinnings of the observed structural changes are and more research in the field of learning‐associated neuroplasticity is needed to incorporate changes in brain function and brain structure into one conceptual framework.

The left IFC, comprising BA 44 and BA 45, plays a critical role in speech processing [Travis et al., 2013], speech production [Wang et al., 2014] and lexical decision making [Crepaldi et al., 2013], but has also been shown to play a critical role in LOPs [Kapur et al., 1994; Otten et al., 2001; Schott et al., 2013], such that tasks involving semantic analyses (deep processing of written words) elicit specific brain activation in the left IFC. The semantic task in this study comprises various aspects of lexico‐semantic processing, e.g. letter assembling, word composition, and lexical decision making. Heim et al. [2009], using fMRI and effective connectivity analyses, could demonstrate that BA 44 and 45 within the IFC have strong intrinsic connections and that BA 45 is modulated by the OTC in both phonological decision as well as in lexical decision tasks. Interestingly in our study neural activity in the OTC was elicited by both words and nonwords alike, while the activation of the IFC seemed to be predominantly elicited by words. In this regard it is tempting to hypothesize that, after sound‐to‐letter conversion within the left OTC, word composition and lexical decision making strongly rely on the left IFC and its interaction with the left OTC. Our current analyses do not allow an exact attribution of these various functions to the specific regions or their interactions, e.g. bottom up vs. top down modulation. This will need to be addressed in future studies both investigating interim steps, such as letter translation (without word translation), and applying more sophisticated analyses such as effective connectivity analyses, to assess the interactions between various brain regions.

## LEARNING AND CORTICAL PLASTICITY

In this study we also sought to investigate cortical plasticity‐associated learning and its relation to different LOPs. For lexico‐semantic processing, we report specific changes in brain activation following learning, with new regions being recruited, i.e. in the left IFC and the left OTC, concurrent with a cognitive shift, which together fulfill the criteria of reorganization (proper), as suggested by Kelly and Garavan [Kelly and Garavan, 2005]. Importantly, the activation pattern flexibly adapts to the LOP, such that the perceptual analysis relies on the perception network, showing strong activation in the STG with no activation in the IFC or OTC. This has important implications for learning and learning‐associated neuroplasticity. First, learning leads to a functional extension of a core network, recruiting new brain regions that can “handle” specific task requirements. Second, depending on the study instructions (perceptual vs. lexico‐semantic), these new brain regions are activated or are not activated, implying that study participants switch between network extensions. Importantly, the perceptual network contains brain regions that are not (or at least to a significantly lesser degree) part of the lexico‐semantic network, implying that lexico‐semantic analysis is not just additive to perceptual analysis, but that the two types of analyses rely on functionally distinct networks, despite a common underlying core.

For the perceptual task, subjects are likely to optimize performance by focusing on a certain property of the stimulus, e.g. tone length. This focus is reflected in brain activation in the STG bilaterally, which makes it conceivable that different mechanisms such as signal amplification and/or short term memory processes contribute to tone perception and length discrimination. Although the assessment of tone length is also relevant to the lexical part, the decision “equally long” or “unequally long” is not. Quite the contrary, such a focus and the corresponding brain activation likely interfere with the lexico‐semantic task. Unlike the perceptual task, the lexico‐semantic task requires linking a sound pattern to a memory trace (lexical access) eliciting an additional information flow from the core network to the left OTC and ITC. What exactly causes the recruitment of specific task‐relevant brain regions remains unclear. On a behavioral level, engaging in a specific LOP requires an attentional focus on specific stimulus features and the negligence of others. On the neural level, specific attention to certain features might lead to pre‐activations in task‐relevant brain regions, subsequently allowing for the recruitment of these regions by the core network.

## LIMITATIONS

There are some limitations to our study that need to be addressed. First of all, our lexico‐semantic task is rather complex and involves several aspects, i.e. a lexical part (sound‐to‐letter conversion), as well as a semantic part (the decision whether a specific three‐letter‐train makes up a word, a nonword or the SOS signal). In future studies participants should also perform a purely lexical task, where they have to decide whether the first and the last letter (in a three‐letter‐train) are the same or not, which will help to further disentangle how specific regional brain activation contribute to different aspects of the current task.

MC in our task involves both the phonological as well as visual letter representation systems. During training (six sessions outside the scanner) study participants would write down the letter after hearing the sound pattern, which served the goal to easily asses performance afterwards. By doing this, phoneme‐to‐grapheme conversion (or sound pattern‐to‐grapheme conversion) and the related brain activation might have become part of the analysis strategy. In this study we cannot control for this potential effect. Future studies will need to investigate whether pure acoustic learning (without writing down the letters) has an effect on performance and related brain activation.

Finally, when comparing the perceptual and the lexico‐semantic tasks, there was a difference in the complexity of the response option. In the perceptual tasks, subjects responded by two fingers indicating whether the first and the last MC signal was the same or not. In contrast, subjects were asked to decide between four response options in the lexico‐semantic task (involving four fingers). Responses were given with the left hand. While we cannot rule out that some differences in brain activation, e.g. in the right premotor/motor cortex (A4‐b, right hemisphere) might indeed be related to differences in complexity of the motor task, we are confident that specific activations in language‐sensitive areas in the left hemisphere are rather related to the LOPs and/or the learning process. However, future studies should adjust response options in both tasks to rule out this potential confound.

## CONCLUSIONS AND OUTLOOK

In summary we suggest that different LOPs, as investigated in this study, rely on a mutual core network, and that learning leads to a functional extension of this network. However, depending on the task requirement, specific activations are seen either in the primary sensory cortices (perceptual analysis) or in the newly‐involved higher association cortices (lexico‐semantic analysis). As such, the transition from perceptual to lexico‐semantic stimulus analysis is accompanied by a shift in brain activation. This shift, however, is flexible and adapts to the LOP demanded by the task. To our knowledge, this is the first study that has investigated both learning‐associated neuroplasticity and neural correlates of LOPs within one study design, demonstrating dynamic network extensions over time, with a flexible, LOP‐related recruitment pattern, a process one might refer to as “adaptive neuroplasticity”.

Morse Code is a highly interesting and flexible tool to investigate learning‐associated neuroplasticity and neural correlates of LOPs. In this study participants learned 12 letters, which required less than 2 weeks and provided a well‐controlled learning environment with respect to preexisting knowledge and practicing time, and, most importantly, elicited robust and reproducible brain activation. In future studies this paradigm will also allow the investigation of more complex aspects of brain activation, such as effective connectivity, which will be essential in determining the influence of one brain region upon another, i.e., the direction of information flow. In our scenario, for example, the interaction of the OTC and IFC needs to be further investigated to disentangle bottom up vs. top down modulation. It is conceivable that the OTC, as hypothesized, is critically involved in sound‐to‐letter conversion, while the IFC deals with letter assembly and semantic decisions. On the other hand, in the conceptual framework of the Bayesian brain and predictive coding approaches, the IFC as a higher association cortex might impose a set of predictions on the OTC (predictive processing), exerting a top down modulation. For this kind of analysis both perceptual and lexico‐semantic stimuli need to be presented within one run, with cues indicating the type of analysis to be performed for the upcoming stimulus. Finally, additional LOPs can be added to the paradigm, e.g. intermediate LOPs where study subjects have to make decision with regard to the sound pattern that compose single letters (not words). This would help to investigate more precisely the roles of the IFC and OTC in sound pattern analysis and sound‐to‐letter conversion without the additional task of word composition. As such, further studies are required to disentangle neural correlates of different LOPs and the learning‐induced mechanisms that allow the transition from one level to another.
